# Challenges and Management of Capillary Intraosseous Hemangioma in the Mandibular Symphysis: A Case Report

**DOI:** 10.7759/cureus.58035

**Published:** 2024-04-11

**Authors:** Érica Cerqueira, Sofia Correia, Fátima Ramalhosa, Teresa Lopes, João Mendes Abreu

**Affiliations:** 1 Maxillofacial Surgery, ULS Coimbra, Coimbra, PRT; 2 Pathology, Clinical and Academic Centre of Coimbra, Coimbra, PRT; 3 Faculty of Medicine, Clinical and Academic Centre of Coimbra, Coimbra, PRT; 4 Stomatology, Clinical and Academic Centre of Coimbra, Coimbra, PRT

**Keywords:** vascular neoplasm, surgical excision, intraosseous, intraosseous hemangioma, capillary hemangioma

## Abstract

Capillary intraosseous hemangioma is a benign vascular neoplasm that affects bone tissue, yet its occurrence in the jaw bones has been seldom reported in the literature.

We present a case of a capillary intraosseous hemangioma located in the mandibular symphysis of a 28-year-old male.

Initially addressed by the patient’s dentist as an infectious lesion of endodontic origin, the sudden worsening of the condition, marked by the development of a rapidly expanding exophytic mandibular lesion and tooth mobility, led to the consideration of various potential diagnoses.

Subsequently, an incisional biopsy was performed, triggering multiple episodes of recurrent bleeding, leading to several visits to the emergency department, and prompting an urgent status upgrade for the patient.

Upon the histological diagnosis of vascular neoplasm, the patient underwent the excision of the lesion, with a favorable and uneventful evolution, although with expected sequelae. As a result, a temporary prosthetic solution, comprising a Maryland Bridge, was implemented, with plans for guided bone regeneration and implant-supported fixed dental prostheses currently in progress.

This case underscores the diagnostic and therapeutic challenges associated with this rare condition. Consequently, achieving the optimal outcome for the patient largely depends on a multidisciplinary approach, emphasizing the critical importance of thorough preoperative assessment, along with a well-devised treatment plan and rapid intervention.

## Introduction

Hemangiomas are benign vasoformative neoplasms of endothelial origin [1,2]. Commonly experiencing a swift postnatal expansion, these lesions then to a spontaneous and gradual regress over several years [1].

Intraosseous hemangiomas are an extremely rare subtype of hemangiomas, constituting approximately 1% of all intraosseous tumors [1-6]. The peak incidence of said variant occurs in the second and fifth decades of life with a female-to-male ratio of 2:1 [1-3,7-9], primarily targeting the vertebral bodies and often manifesting itself asymptomatically [1,3,6].

Although rare in the mandible [1-3,7-9], symptomatic cases may exhibit signs such as a slowly enlarging bluish mass, a pulsating sensation, gingival bleeding, and tooth mobility [1-3,9,10]. Moreover, episodes of severe bleeding can arise, triggered by trauma such as dental extraction or biopsy of the lesion, which can result in significant complications [2,5,7,8,10,11]. More rarely, these lesions can exhibit an aggressive behavior, characterized by rapid growth and osteolytic (destructive) capabilities [3,4,8].

Panoramic radiographs, computer tomography (CT) scans and magnetic resonance imaging (MRI) are the most useful radiographic studies when considering intraosseous hemangiomas [1,2]. Radiological findings are diverse but most frequently present a multilocular radiolucent image with a *honeycombed* or *sunburst* appearance [1-3,6].

Although different therapeutics are available, surgical excision is the gold standard for intraosseous hemangiomas [1,4,6,10]. Nonetheless, it's important to note that, in most cases, treatment is not deemed essential, and the prognosis remains excellent [12].

This report aims to present a case of an exceptionally rare central mandibular hemangioma and discuss the challenges associated with its diagnosis, given the variability in clinical and radiological presentations.

## Case presentation

A 28-year-old male with an unremarkable medical history and no current medications was referred to the Emergency Department for an exophytic central mandibular lesion.

The patient affirmed that the lesion was primarily identified by his dentist and diagnosed as an infectious lesion of endodontic origin. Subsequently, the initial course of action included proposing and executing root canal treatment on teeth 31 and 32.

However, during the following weeks, the patient reported no improvement and a subsequent worsening of the lesion. The patient also negated experiencing pulsatile sensations, bleeding, or discomfort.

**Figure 1:**
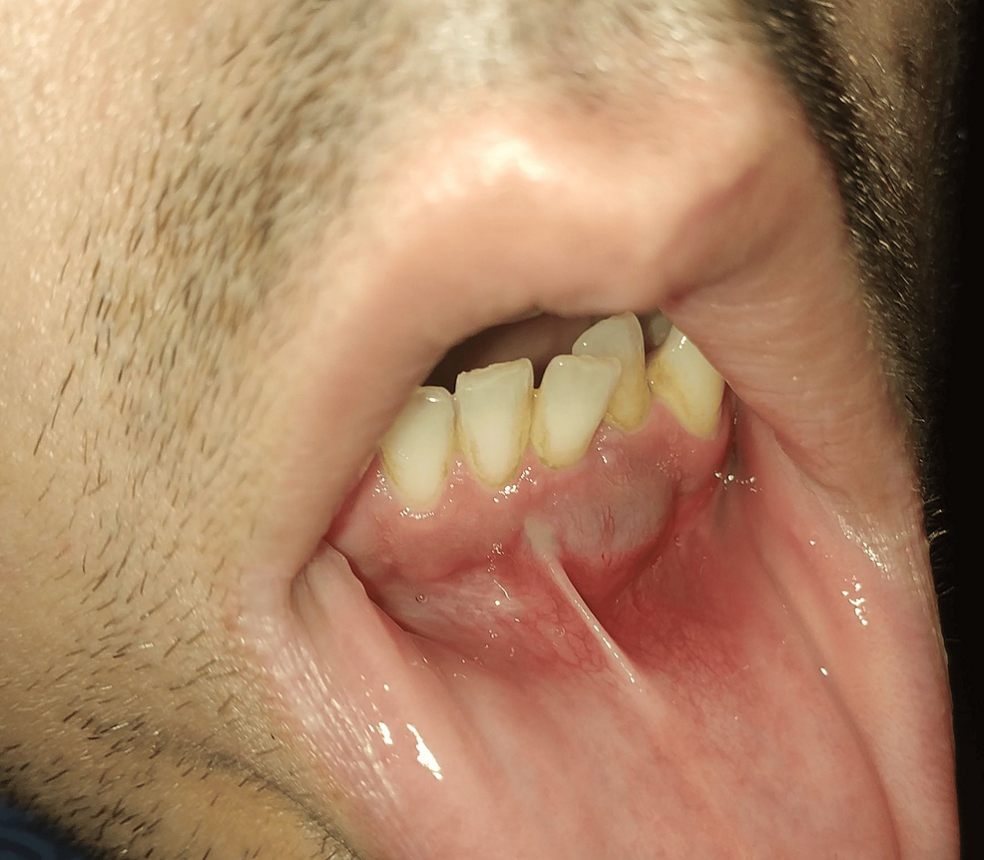
Mandibular mass: visible in an intraoral view, a nodular mass, approximately 15 mm in width, located in the left anterior portion of the mandible.

Upon intraoral examination, a blue-red mass, measuring approximately 15 mm, was observed in the left mandible. The mass exhibited an elastic consistency, susceptible to digital pressure, and grade 2 mobility was noted in teeth 31, 32, and 41 (Figure 1).

No pulsation or fremitus was palpated. There were also no detectable cervical or axillary lymph node enlargements upon the exploration of said areas.

**Figure 2:**
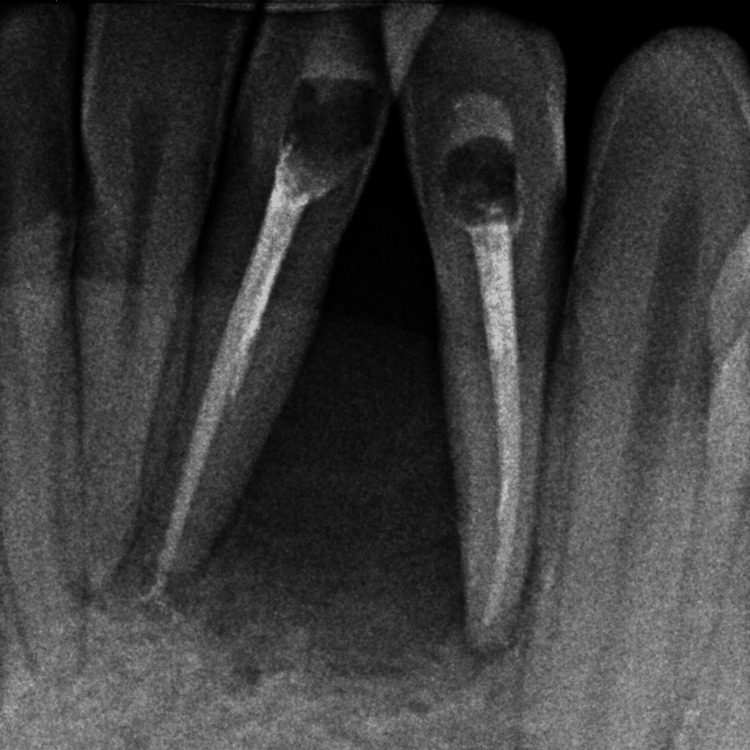
Periapical radiograph showing a rounded well-defined radiolucency and the lateral displacement of teeth 31 and 32.

After the initial evaluation, a digital periapical radiograph was performed revealing a rounded well-defined radiolucency in the mandible, accompanied by the lateral displacement of teeth 31 and 32 (Figure 2).

**Figure 3:**
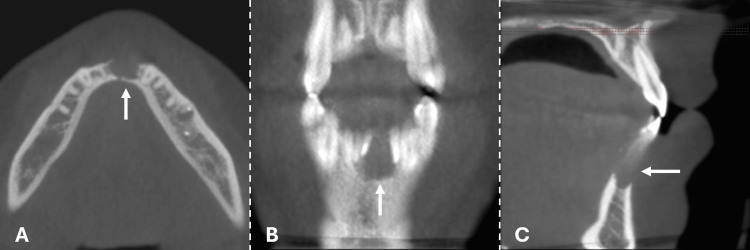
CBCT showing a pre-operatory intraosseous mandibular lesion (arrows): (A) axial, (B) coronal, and (C) sagittal views.

Given the rapid progression of the pathology and the current strain on resources causing extended wait times for CT scans or MRI appointments, it was deemed necessary to opt for a cone beam computed tomography (CBCT) instead. Utilizing the i-CAT CBCT scanner, with specific parameters including a tube current set at 4 mA, a voxel size of 0.4 mm, and a tube voltage of 120 kV, the examination proceeded promptly to address the urgency of the situation. The exam revealed an 8.5 mm unilocular lesion with distinct limits, as well as periosteal reaction and cortical expansion of the mandible (Figure 3).

Despite the lesion causing the distancing of the roots, no signs of resorption were evident.

Considering the clinical presentation and imaging findings, differential diagnoses for the lesion were proposed, encompassing pyogenic granuloma and peripheral giant cell granuloma. Consequently, an incisional biopsy was performed to obtain a preliminary diagnosis.

**Figure 4:**
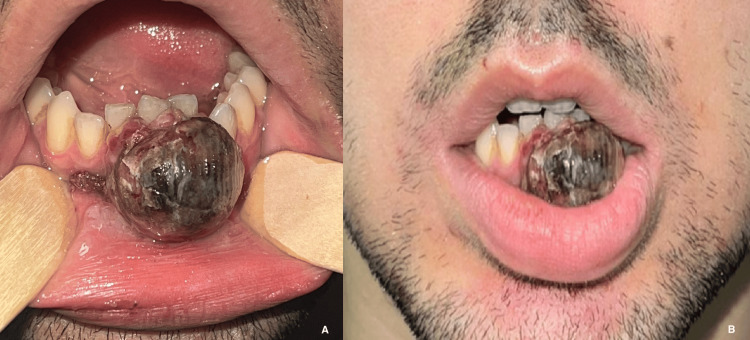
Mandibular mass. Previously reported mandibular mass showing a significant enlargement, conditioning involuntary lip separation: (A) intraoral view and (B) extraoral view.

However, one week after the biopsy, the patient returned to the Emergency Department due to worsening teeth mobility and misalignment, and persistent bleeding from the lesion, which proved unresponsive to local measures. Upon examination, a significant enlargement of the lesion was observed, accompanied by a diminished but continued hemorrhage (Figure 4).

Hemostasis was successfully achieved through the topical application of silver nitrate, intravenous tranexamic acid, and localized cryotherapy with ice packs. This behavior prompted the consideration of bone hemangioma as a diagnostic possibility, expanding beyond the previously entertained hypotheses.

In the meantime, histopathological examination diagnosed the lesion as a neoplasm of vascular nature without features of aggressiveness, classifiable as a hemangioma.

After diagnostic confirmation and considering the rapid growth and occlusal modifications, a prompt intervention was deemed necessary. Consequently, the radical excision of the lesion was proposed and accepted by the patient, which also entailed the extraction of teeth 41, 31, and 32 under general anesthesia.

**Figure 5:**
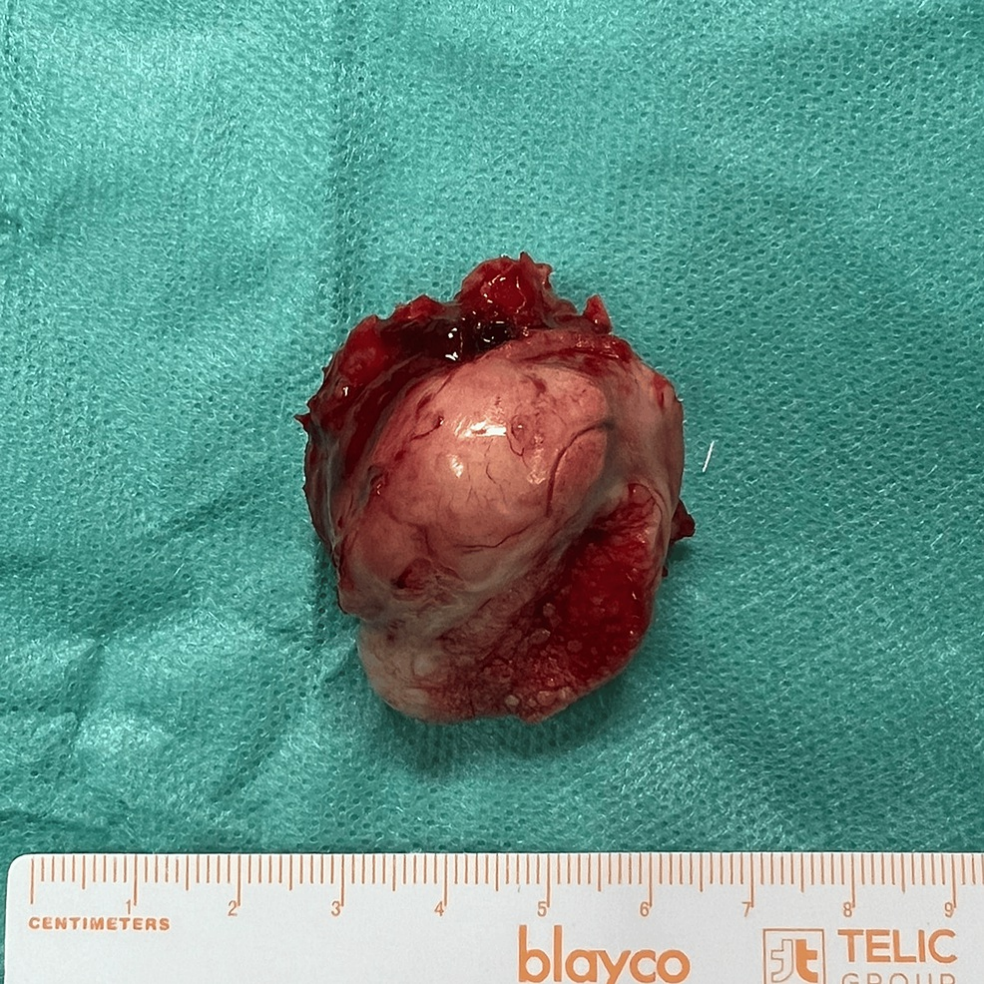
Post-excision view of the mandibular mass.

The surgical specimen was macroscopically characterized as a nodular formation measuring approximately 36 mm wide. It exhibited a mixed surface composed of pink discoloration and congestion, with marked irregularity (Figure 5).

**Figure 6:**
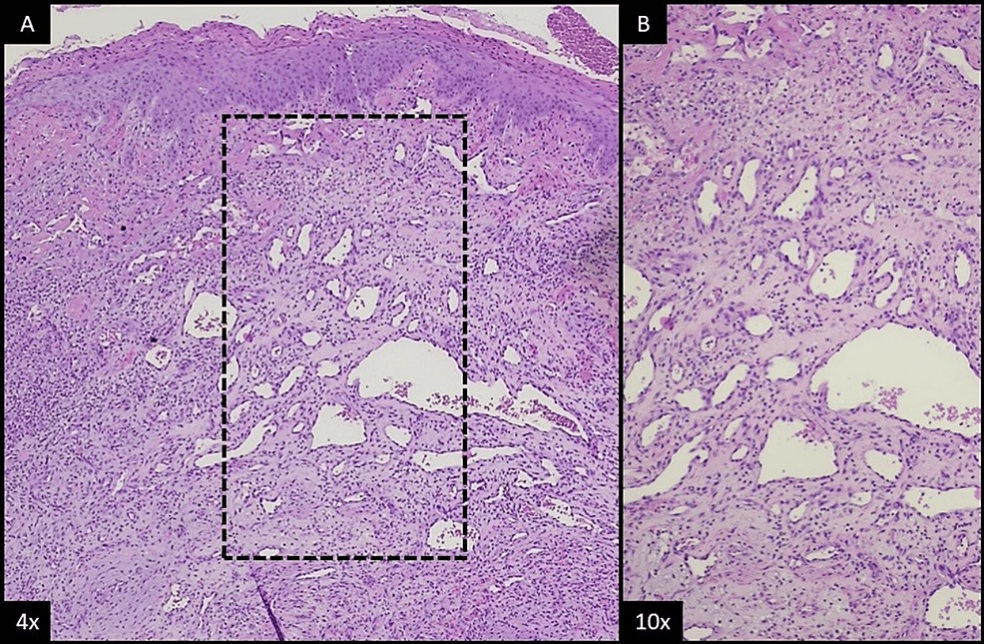
Intraosseous capillary hemangioma the mandible (histological analysis with hematoxylin and eosin staining). (A) At a x4 optical magnification, one can see a mucosal fragment, covered by a thin and partially ulcerated epidermis, with a fibrinous exudate with a mixed inflammatory cell infiltrate. (B) At a x10 optical magnification, in the stroma, a lobular pattern of vascular proliferation of small- to medium-sized vessels can be identified, surrounded by endothelial cells without atypia.

Microscopic examination revealed a vascular proliferation consisting of small capillaries surrounded by normal endothelial cells, devoid of atypia. This established the definitive diagnosis as a capillary hemangioma (Figure 6).

**Figure 7:**
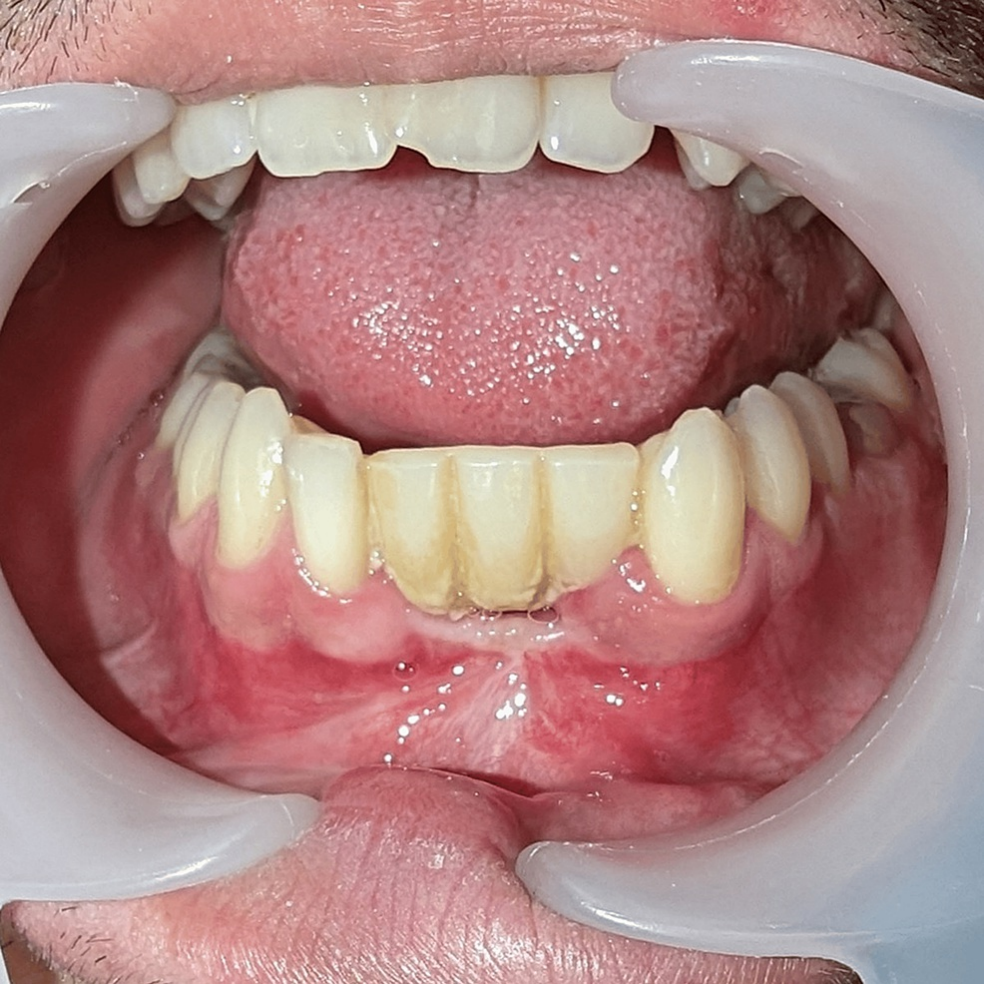
Post-excision status of the intraosseous capillary hemangioma of the mandible (12-month follow-up) Visible in an intraoral view, the surgical site shows the complete healing of the soft tissues without signs of recurrence, although with significant scarring.

After a favorable and uneventful evolution, the patient was discharged within 24 hours. Subsequent follow-up occurred over 12 months, during which the healing process unfolded without complications, and no clinical evidence of recurrence was observed (Figure 7).

**Figure 8:**
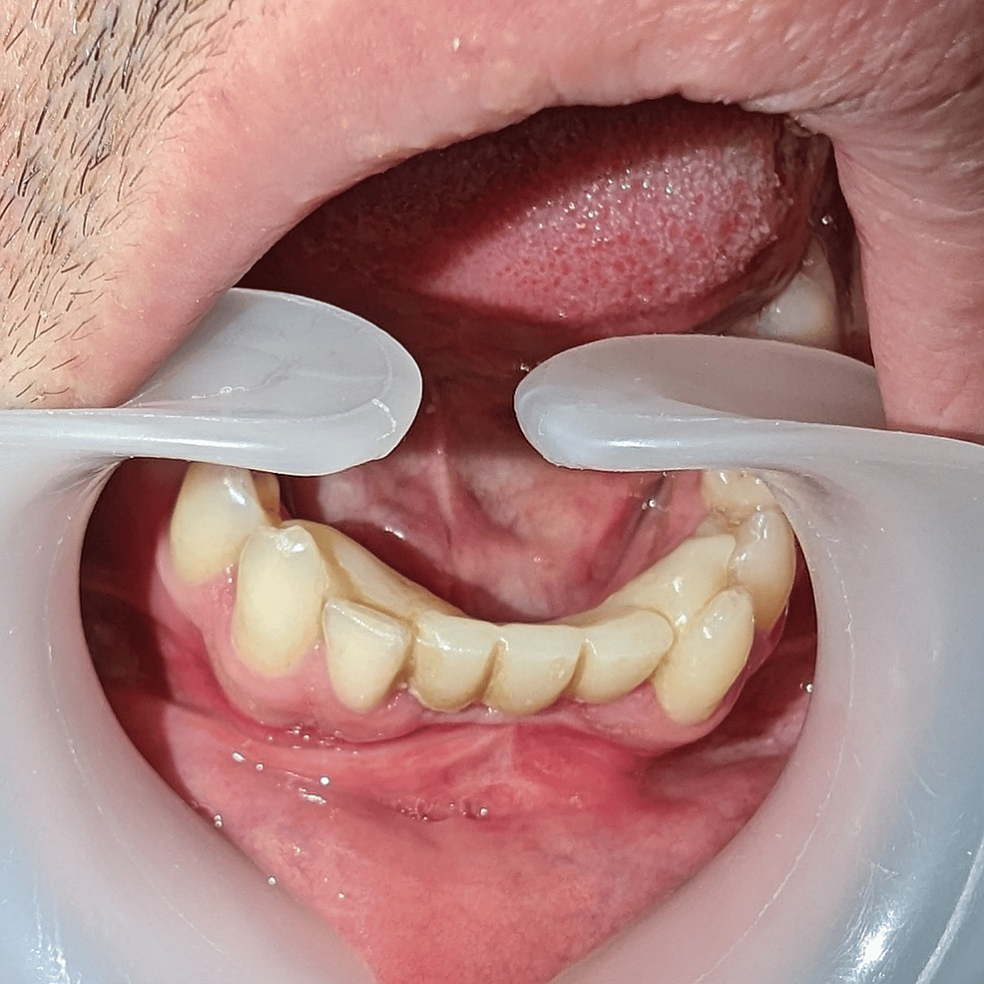
Temporary prosthetic solution consisting of a Maryland Bridge, encompassing teeth 41, 31, and 32.

Throughout this period, the patient was provided with a temporary prosthetic solution consisting of a Maryland Bridge, encompassing teeth 41, 31, and 32, and yielding satisfactory results (Figure 8).

**Figure 9:**
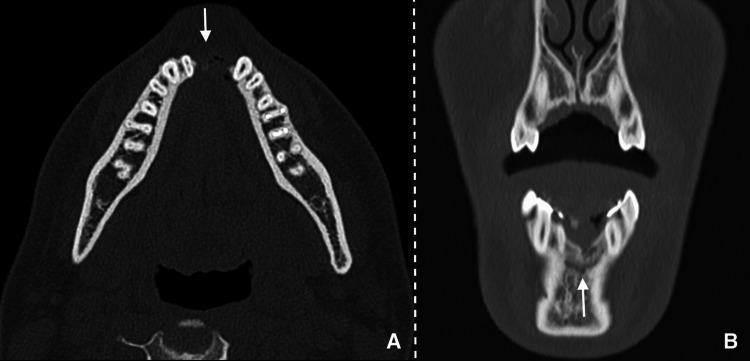
Twelve-month follow-up maxillofacial CT scan showing the defect (arrows). (A) Axial and (B) coronal views. CT, computed tomography

A maxillofacial CT scan was also conducted 12 months post-surgery, revealing favorable bone consolidation (Figure 9).

Henceforth, there is optimistic anticipation for the prospect of performing a guided bone regeneration procedure and subsequent oral rehabilitation of the edentulous space through the utilization of implant-supported fixed dental prostheses.

## Discussion

Hemangioma is a clinical term used to describe a benign vascular neoplasm of endothelial origin. Histologically, based on the type of blood vessels involved, it can be classified as capillary, cavernous, or mixed [13-15].

Intraosseous hemangioma of the jaw is an exceedingly rare condition, and when found in this location, it commonly manifests in its cavernous form. The capillary form is even more uncommon, with only two reported cases [16,17].

Dereci et al. and Kochaji et al. described a 68-year-old woman with a capillary hemangioma in the right and left mandibular body, respectively [16,17]. Both cases were instances of an imaging finding without associated clinical symptoms, contrary to our case report, which shows a capillary hemangioma of the jaw in a young individual with associated disfiguring clinical symptoms.

The pathogenesis of hemangiomas remains up for debate, with several theories proposed to explain their development. Some authors propose that hemangiomas are congenital lesions, while others attribute their development to trauma [2,13,15]. However, no history of trauma or predisposition for the development of intraosseous hemangioma was identified in our case.

Diagnosing this condition can pose challenges, given that patients are typically asymptomatic, and its discovery often occurs incidentally during routine radiographic examinations [14]. Nevertheless, some patients may report symptoms, including pain or discomfort, paresthesia, facial asymmetry, and teeth mobility [15,16]. Initially, the patient's clinical presentation did not align with the typical characteristics of a bone hemangioma. However, the subsequent evolution of the lesion, post-incisional biopsy, exhibiting progressive growth, a bluish mass, recurrent episodes of bleeding, and increasing teeth mobility, led to the consideration of bone hemangioma as the most probable diagnostic hypothesis.

Indeed, the initial diagnosis is often complex since radiographically the characteristics of bone hemangiomas can closely resemble those of other lesions. This overlap in features can make it challenging to definitively identify the condition. These lesions may manifest as a multilocular radiolucency, presenting a honeycomb or soap bubble pattern, or as an unilocular radiolucency [6,15]. CT scan and magnetic resonance angiography (MRA) are also valuable tools for the etiological assessment and staging of the lesion, as highlighted in the literature [13]. Preoperative arteriography is typically deemed unnecessary as, in most cases, no vascular flow can be detected [13]. However, it should be considered in large lesions, along with preoperative embolization, to minimize surgical bleeding [4].

The treatment approach for intraosseous hemangioma varies depending on its presentation. Tailoring the treatment strategy to the specific characteristics of the hemangioma ensures optimal management outcomes by adopting a symptom-oriented approach, thereby restricting therapeutic intervention to a small fraction of cases. Indications for treatment typically take place in cases of aesthetic disfigurement, mass effect, pain, and recurrent episodes of bleeding. Otherwise, a wait-and-see approach is preferred due to the benign nature of these lesions [2,13]. Surgical excision with reconstruction stands as the ideal treatment, while alternative therapeutic modalities such as curettage, radiotherapy, and embolization are also viable options [15]. The utility of preoperative embolization is evident in cases involving large lesions, where its purpose is to minimize surgical bleeding [15].

In this case, due to the rapid growth of the lesion, dental displacement, and repeated bleeding episodes, treatment consisted of the surgical excision and extraction of the affected mobile teeth. Preoperative embolization was not deemed necessary, given the lesion's small size and favorable radiographic characteristics.

The prognosis following complete excision is excellent, and recurrence is typically rare [2]. In the presented case, there was no clinical or radiological evidence of recurrence over a 12-month postoperative period. A maxillofacial CT scan also revealed adequate bone regeneration and consolidation.

During this period, oral rehabilitation was accomplished using a type of resin-bonded fixed partial denture, commonly identified as a Maryland Bridge. A favorable option with a beneficial balance between benefits, risks, and costs serves as an optimal solution in the anterior sector for temporary or even medium-term periods [18]. However, this solution may be deemed insufficient as it impairs the overall function of the replaced teeth and can manifest increased susceptibility to failure when compared to implant-supported fixed dental prostheses [19]. Therefore, considering the patient's age, expectations, economic means, mandibular defect, and bone status, the patient is now cleared to commence vertical and horizontal mandibular augmentation, thereby establishing the necessary conditions for posterior oral rehabilitation with dental implants [20].

The limitations of this study stem from practical constraints inherent within the Portuguese national health system. The decision to opt for a CBCT instead of a contrast-enhanced CT or MRI was primarily influenced by the constrained availability of human and material resources, alongside the shorter waiting times associated. 

Furthermore, the necessity to examine on an outpatient basis due to the patient's failure to meet urgent CT scan criteria adds another layer of limitation. This decision could potentially impact data collection procedures and the overall quality of outcomes. Acknowledging these constraints is essential for obtaining a comprehensive understanding of the study's findings. 

## Conclusions

The rarity of intraosseous mandibular hemangiomas, as evidenced by this study, emphasizes the wide range of clinical, radiographic, and histological presentations they can manifest. This study demonstrates a unique clinical presentation of a mandibular capillary hemangioma, not described in the literature, with symptomatic and defacing clinical presentation. This underscores the significance of recognizing and comprehending the diversity in their manifestations.

Consequently, it is crucial to note that these patients may encompass small, asymptomatic lesions as well as aggressive, osteolytic, larger lesions carrying a potential risk of bleeding, as presented in this case. Hence, it is of the utmost importance that, in managing such patients, a thorough preoperative assessment and a well-devised treatment plan are elaborated and implemented.
